# Mental Resilience of Medical Practitioners in Singapore during COVID-19: Survey Results from a Webinar Course on Resilience

**DOI:** 10.3390/ijerph18189801

**Published:** 2021-09-17

**Authors:** Cheng Hong Charity Low, Tze Jui Goh, Yiong Huak Chan, Daniel Shuen Sheng Fung, Pak Yean Cheong

**Affiliations:** 1 Peace Family Clinic (WL 832), Block 832, #01-71, Woodlands Street 83, Singapore 730832, Singapore; charitylow@yahoo.com; 2 Institute of Mental Health, Buangkok Green Medical Park, 10 Buangkok View, Singapore 539747, Singapore; daniel_fung@imh.com.sg; 3 Yong Loo Lin School of Medicine, National University of Singapore, 1E, Kent Ridge Rd, NUHS Tower Block, Level 11, Singapore 119228, Singapore; medcyh@nus.edu.sg (Y.H.C.); pakyean@gmail.com (P.Y.C.)

**Keywords:** COVID-19, general practitioners, zones, fear, learning, growth

## Abstract

Front-line doctors are at high risk of exposure to COVID-19. The mental resilience of general practitioners and their areas of concerns and support required are important during this COVID-19 period. A total of 403 general practitioners attending a webinar on resiliency, hosted by the College of Family Physicians, Singapore, participated in the survey anonymously. Participants provided responses to questions relating to COVID-19 in the domains of *Family and friends*, *Myself*, *Practice*, and *Community*. Responses are categorized into LEARNING, FEAR, and GROWTH zones. The majority of the doctors reported to be in the GROWTH zone in relation to *Family and friends* (39%) and *Myself* (38%) as compared to *Practice* and *Community*, *a*OR = 4.5 (95% CI 3.4 to 5.9), *p* < 0.001. 34% of the participants reported being in the FEAR zone in relation to *Family and friends*, *a*OR = 8.0 (95% CI = 5.6 to 11.2), *p* < 0.001; at least 81% reported being in the LEARNING zone in relation to *Practice* and *Community*, *a*OR = 7.5 (95% CI = 5.8 to 9.6), *p* < 0.001, compared to other domains. Supporting and protecting the doctors is important in strategic planning and management of the current pandemic and building preparedness and an effective response towards future crises.

## 1. Introduction

COVID-19 has swept through the globe with devastating effects since 2020, with no sign of abating to date. The World Health Organization (WHO) has reported over 140 million confirmed cases of COVID-19, with more than 3 million deaths [1]. About 10% of these cases are front-line healthcare workers [2], who have been reported to be 3.4 times at a higher risk of contracting COVID-19 in comparison to the general community [3]. Globally, at least 1800 healthcare workers were reported to have died of COVID-19 [4]. As of 19 April 2021, there were 60,831 confirmed cases and 30 deaths in Singapore [5].

General medical practitioners make up 80% of primary care (private or public community) services in most healthcare systems and are prominently at the front-line. They are, commonly, the first touchpoint of cases presenting with early upper respiratory tract infection (URTI) symptoms. In Singapore, about 900 private general practitioner clinics were activated as Public Health Preparedness Clinics (PHPC) starting 18 February 2020 as part of the nation’s fight against the pandemic. Patients with URTI symptoms were encouraged to seek treatment at these clinics, with subsidies provided by the government to cover the cost of testing for COVID-19. General practitioners at these clinics had prior training in infection control measures and were provided the necessary resources such as personal protective equipment (PPE) and test kits. They receive patients who present with URTI symptoms, provide medical treatment accordingly, as well as conduct surveillance testing of COVID-19. When a COVID-19 positive case is detected, the GPs contact the relevant agencies to transport the patient to an identified isolation facility for further treatment and management. The GPs are also provided follow-up support such as disinfection of the premises, to contain possible transmission of the virus. Evidently, the timely intervention and management of potential COVID-19 cases by the GPs is fundamental in the successful early detection, isolation, management, and prevention of widespread transmission of COVID-19. Hence, the mental state of general practitioners in response to an infectious disease outbreak, especially to one on such a massive scale as the COVID-19 pandemic, is of great concern. There is a consensus in studies examining the well-being of doctors during COVID-19 that doctors are at higher risk of suffering from an acute stress reaction, burn-out, insomnia, anxiety, depression, and post-traumatic stress disorder, compared to the general population. These have long-term psychological implications [6,7]. Resilience is a multi-factorial concept, usually framed as avoidance of burnout in the context of workplace stress. However, aside from stress management or distress tolerance strategies, resiliency in health professions likely involves other factors such as adaptation skills and personal resources such as professional identity, support from family, peers, and the professional community [8]. Few studies have focused on the resilience of doctors in times of crisis. While some studies reported that task-oriented management was preferred by general practitioners during the COVID-19 crisis [9], it is likely that different strategies could be necessary at different phases in the coping of the pandemic. A balance of individual and organization strategies, including psychological intervention or support programs, could be useful in mitigating the psychological impact of crises on the well-being of healthcare workers [10].

In this study, resilience is conceptualized as a continuum of mental states ranging from fear to growth, in an individual’s response to crisis. Fear, as an instinctive emotional response, is fundamental to the survival of the self [11] and can manifest as anger, sadness, or anxiety. In times of stress, an individual may cope with the fear response by focusing on the protection of the self at the expense of others, or via externalizing of problems, i.e., finding blame, or responding with withdrawal or denial, or engaging in hyper-response to triggers. While the functional value of these responses may vary with external circumstances, resources, and environment, the choice of response also reflects on the internal resiliency of the individual. One may be successful in overcoming the initial fear response and developing a learning attitude and approach towards crisis management. Learning can be seen as accepting the situation; and developing adaptive responses to problem-solve and cope with the changes accompanying the crisis. Growth is a phase whereby the individual is able to creatively extend beyond the problem-solving stage, and acquire new skills, thrive, and rise above the existing challenges to make plans and consider future goals. We encapsulate these as ‘zones’ i.e., FEAR zone, LEARNING zone, and GROWTH zone, in the conceptualization of resiliency.

Given that the general practitioners are likely to experience greater distress during the COVID-19 pandemic, we assess the prevailing mentality of doctors during COVID-19 using the ‘zones’ in resiliency by determining which ‘zone’, i.e., FEAR, LEARNING or GROWTH zones, they are in, with reference to the domains of *Myself*, *Practice*, i.e., work setting, their *Family and friends*, and the *Community* at large. The study further examines the impact of age group, area of practice, and the risk of exposure to COVID-19 on the mentality of the doctors.

## 2. Method

General practitioners attended a Webinar titled: *Resilience in Times of COVID-19 for Frontline Doctors I*, hosted by the College of Family Physicians, Singapore (CFPS), on 3 May 2020 [12]. The webinar was the first of a set of four seminars conducted after clinical hours from 9.00 to 10.30 pm (SGT) whereby the GPs tuned in to learn about the latest findings, practice, insights about the COVID-19 situation, as well as learn about and discuss about mental well-being issues and coping strategies for themselves as they continued their practice during this stressful period. Such training and workshops are part of the continuing medical education organized regularly by the CFPS. Participants were invited to participate in the anonymous survey (refer to Supplementary Materials) during the break time of the webinar. The study was approved by the National University of Singapore Institutional Review Board (Ref: NUS-IRB-2020-105).

### 2.1. Participants

A total of 403 out of 504 (80%) participants responded to the survey. All participants were medical practitioners licensed with the Singapore Medical Council.

### 2.2. Measures

**Figure 1:**
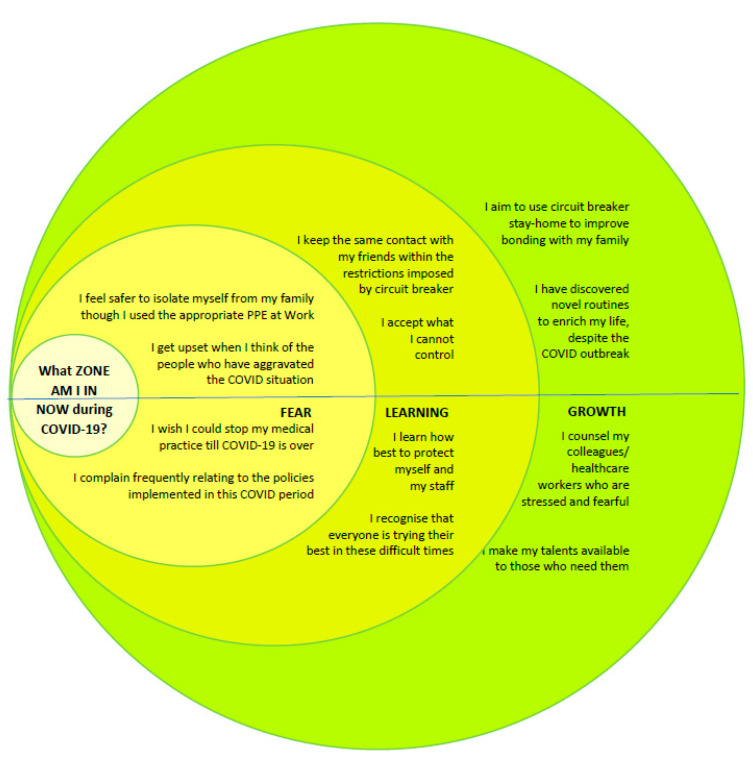
What zone am I in now during COVID-19?

Respondents were asked to respond to the survey (Annex 1) administered electronically via the webinar interface. Demographic data on their age group, and work setting (place of practice), and experience of exposure to COVID-19, were collected. Participants also responded by choosing one out of three statements that corresponded to the ‘zones’ in resiliency, relating to (1) Family and friends, (2) Myself, (3) Practice/work setting, and (4) Community. The responses corresponded to the FEAR, LEARNING, and GROWTH zones (Figure 1).

### 2.3. Data Analyses

All analyses were performed using SPSS for Windows version 26.0 (SPSS Inc., Chicago, IL, USA) with statistical significance set at 2-sided *p* < 0.05. Descriptive statistics for the categorical responses were presented as *n* (%). Associations of the socio-demographical variables with the three main Zones (FEAR, GROWTH, and LEARNING) were assessed using logistic regression.

## 3. Results

### 3.1. Sample Characteristics

Of the 403 respondents, the majority (*n* = 258; 64%) were aged 45 to 65 years old, working in private practice (*n* = 311, 77%). Almost two-thirds (64%) of the participants had encountered a suspected COVID-19 case, of which more than half of them (34%) were confirmed positive. Table 1 presents the detailed demographic characteristics of the participants.

### 3.2. Main Analyses

Table 2 shows that 34% of the participants responded in the FEAR zone in the domain of *Family and friends,* compared to the other domains (ranging 4% to 12%), *a*OR = 8.0 (95% CI 5.6 to 11.2), *p* < 0.001, adjusting for age, practice, and exposure to COVID-19. At least 81% responded in the LEARNING zone in the domains of *Practice* and the *Community* compared to 27% and 50% for the domains of *Family and friends* and *Myself* respectively, *a*OR = 7.5 95% CI 5.8 to 9.6), *p* < 0.001. Most of the doctors (38% to 39%) responded in the GROWTH zone for the domain of *Family and friends* and *Myself,* compared to 14% for the domain of *Practice* and the *Community*, *a*OR = 4.5 (95% CI 3.4 to 5.9), *p* < 0.001.

### 3.3. Impact of COVID-19 Exposure

#### 3.3.1. Domain: Family and Friends

Participants with ‘No COVID-19′ encounters (49.7%) were more likely to be in the GROWTH zone compared to participants who encountered ‘Positive outpatient COVID cases’, *a*OR = 2.2 (95% CI 1.3 to 3.8), *p* = 0.002; and compared to those who encountered ‘Suspected, later negative cases’, *a*OR = 1.8 (95% CI 1.1 to 3.0), *p* = 0.032. Participants who encountered ‘Positive outpatient COVID’ cases (50.0%) were nearly twice as likely to be in the FEAR zone compared to participants with ‘No COVID-19′ encounters (28.0%), *a*OR = 1.8 (95% CI 1.1 to 3.0), *p* = 0.027.

#### 3.3.2. Domain: Myself

Participants who encountered ‘Suspect, later negative’ cases (18.6%) compared to ‘Positive outpatient COVID’ cases (6.6%) were more likely to be in the FEAR zone, *a*OR = 3.5 (95% CI 1.5 to 8.0), *p* = 0.003.

#### 3.3.3. Domain: Practice/Work Setting

Those who encountered ‘Positive inpatient COVID-19′ cases (33.3%) were more likely to be in the FEAR zone compared to those with ‘No COVID-19′ encounters (5.6%), *a*OR = 15.1 (95% CI 1.01 to 227.0), *p* = 0.049; and those who encountered ‘Positive outpatient COVID-19′ cases (2.2%), *a*OR = 61.2 (95% CI 3.6 to 1047.3), *p* = 0.005 and those who encountered ‘Suspect, later negative’ cases (4.2%), *a*OR = 26.1 (95% CI 1.7 to 407.4), *p* = 0.002.

### 3.4. Impact of Practice/Work Setting

#### 3.4.1. Domain: Family and Friends

There were significantly more participants working in the ‘Others’ setting (57.7%) in the GROWTH zones compared to in the ‘Public community’ (25.8%), *a*OR = 4.0 (95% CI 1.3 to 12.6), *p* = 0.019.

#### 3.4.2. Domain: Myself

Participants in ‘Others’ setting (53.8%) were three times more likely to be in the GROWTH zone compared to participants working in the ‘Hospital’ (25.7%), *a*OR = 3.1 (95% CI 1.02 to 9.6), *p* = 0.045.

#### 3.4.3. Domain: Community

Participants in ’Others’ setting (92.3%) compared to participants working in the ‘Hospital’ (68.6%) were more likely to be in the LEARNING zone, *a*OR = 5.4 (95% CI 1.03 to 28.4), *p* = 0.045. Participants working in the ‘Hospital’ (28.6%) were more likely to be in the GROWTH zone compared to participants in the ‘Others’ setting, *a*OR = 3.5 (95% CI 1.5 to 8.4), *p* = 0.035, and compared to participants working in the ‘Private’ setting, *a*OR = 6.0 (95% CI 1.1 to 31.6), *p* = 0.005. Table 3 shows the distribution of responses to individual questions by area of practice in each domain.

### 3.5. What Zone Am I in Now?

The doctors were classified into a particular zone if three of their responses across the domains fell into the particular zone. Five participants were in the FEAR zone (1.2%). The majority of the participants (*n* = 202, 50.1%) were in the LEARNING zone, while 29 (7.2%) participants were in the GROWTH zone. A significant proportion of participants, *n* = 167 (41.4%), were in the MIXED zone i.e., scored 2 or less than 2 in different zones.

Participants aged 45–64 years (57.2%) compared to 35–44 years (37.5%) were more likely to be in the LEARNING zone, *a*OR = 2.0 (95% CI 1.02 to 3.8), *p* = 0.043. Participants aged 35–44 years (56.3%) compared to 45–64 years (37.2%) were more likely to be in the MIXED Zone, *a*OR = 2.4 (95% CI 1.3 to 4.6), *p* = 0.008. Participants working in the ‘Others’ setting (19.2%) were more likely to be in the GROWTH zone compared to those working in ‘Private’ settings (5.1%), *a*OR = 4.7 (95% CI 1.5 to 14.5), *p* = 0.008. Table 4 shows the overall distributions of responses in each zone based on age groups and area of practice.

## 4. Discussion

The distribution of the participants, with the majority of them in the LEARNING zone, suggested that the general practitioners were generally coping well with the pandemic. The result is encouraging, given that the study was conducted during the initial acute phase of the pandemic when the cumulative number of confirmed cases in the community was increasing daily. Based on the responses from our sample, the probability of encountering a positive COVID-19 case is relatively high at 34%, i.e., about one in three patients. Our study provides some insight into the mentality of general practitioners during a disease outbreak and highlights areas to target for improvement.

The low sense of FEAR amongst the medical practitioners in our sample could be attributed to their trust in the medical system, as well as their confidence in the management of the situation by the authorities locally. While some studies reported fear amongst healthcare workers, attributable to shortage of PPE supply, poor quality or lack of masks, and overwhelmed medical services [13,14], these did not occur in the local setting. Open and updated communication from the authorities, the availability of basic medical resources such as masks and PPEs and the timely activation of additional resources such as the intensive care unit facilities in the National Centre for Infectious Diseases, clear and strict guidelines implemented nationwide, provision of subsidy for patients with acute respiratory symptoms to encourage the timely seeking of treatment, as well as other revised work processes adjusted to the pandemic situation, likely instilled confidence in the medical community and allayed fears within the local healthcare services [15].

Aside from the low levels of FEAR overall, it is interesting that most of the participants responded in the GROWTH zone in the domain of *Family and friends* compared to the LEARNING zone for the other domains. The priority of medical practitioners and the source of support they draw from as they assume their roles in managing the pandemic cannot be over-emphasized. Familial support is likely an important factor in the coping and fulfillment of their doctor role during the COVID-19 outbreak [16,17]. In contrast, healthcare workers have reported experiences of stigmatization (e.g., avoidance of contact or shunned) by members of their community amid concerns that they would be sources of infection [18,19,20]. However, such occurrence is likely less apparent in the local context with social movements and ground-up initiatives organized to support and show appreciation to healthcare workers. These could explain the low levels of FEAR overall in our sample. Nonetheless, understanding and support from family and friends can serve as protective factors that are pivotal to enhance ‘doctors’ resilience in combating the stigma and continue to treat patients [21]. On the other hand, a lack of social support could result in detrimental effects on the mental well-being of the doctors [22,23].

Participants in the ‘Private’ setting appeared to report more FEAR compared to the other settings, such as those in the public hospital setting who may be at a higher risk of exposure to COVID-19 cases. While we are not able to derive causal relations, we can postulate that it may reflect on the overall sense of support mitigated by the perceived level of risk experienced by private practitioners as compared to those in other settings. An increased sense of LEARNING or GROWTH in these settings could also be facilitated by having the GPs work in teams, and ensuring timely communication of information about the situation. The inclusion of ‘family and friends’ as valuable domains of support and the provision of training, are factors that can better support and build up resiliency. Going forward, the introduction of a support system can be considered in the improvement of resiliency and coping levels of first responders and front-line workers in pandemic or crisis management.

The study is not without limitations. While it provided a glimpse into the mental state of general practitioners during the height of local community transmission of COVID-19, the study was descriptive and naturalistic, and we are not able to derive causal relationship. The study was also conducted swiftly in response to the escalating situation of COVID-19 in Singapore, during a time when there was no available tool calibrated for the specific crisis. Among the respondents providing first-hand feedback on their coping and concerns, however, there may be inherent sampling bias as participants had proactively registered for the COVID-19 related webinar. These participants may have been more likely to be in the ‘LEARNING’ zone given their propensity to actively participate in CME workshops and trainings. Given the anonymity nature of the survey, we were also unable to verify detailed demographic information or ensure the accuracy of the responses. The brevity of the survey also limited the reliability of our findings. Specifically, our findings may not be generalizable to other regions with differential response and management of the COVID-19 pandemic. Given that the pandemic is still ongoing, further studies could examine the longer impact of the pandemic on the resiliency of the general practitioners and factors that could enhance the well-being of the healthcare community in times of emergency. Analysis of such information is important in strategic planning to manage the current pandemic and future crises.

## 5. Conclusions

Our results suggest that the majority of doctors were in the GROWTH zone in relation to *Family and friends* and *Myself*, compared to *Practice* and *Community*. At least 81% reported being in the LEARNING zone in relation to *Practice* and *Community*, which is an encouraging outcome, and points to a high level of resiliency in the doctors, especially given that the survey was conducted during the height of local community transmission of the virus. The positive mentality of the doctors could be considered in the broader context of the prevailing management of the pandemic in the local setting. A supportive environment and sense of security and trust in the effectiveness of crisis management by the local authorities likely contributed to positive mental health in the doctors at the front-line. The resiliency and capability of the general practitioner community is an essential part of preparedness in global pandemic response, given that COVID-19 is unlikely to be the last infectious diseases outbreak we see.

## Figures and Tables

**Table 1:** Demographics.

Age	Area of Practice, *n* (%)				Total, *n* (100%)
	Private Community	Public Community	Hospital	Others	
34 years and below	20 (5.0%)	4 (1.0%)	6 (1.5)	1 (0.2%)	31 (7.7%)
35–44 years old	32 (7.9%)	7 (1.7%)	8 (2.0%)	1 (0.2%)	48 (11.9%)
45–64 years old	209 (51.9%)	15 (3.7%)	19 (4.7%)	15 (3.7%)	258 (64.0%)
65 years and above	50 (12.4%)	5 (1.2%)	2 (0.5%)	9 (2.2%)	66 (16.4%)
Total	311 (77.2%)	31 (7.7%)	35 (8.7%)	26 (6.5%)	403 (100%)

**Table 2:** Distribution of domains and COVID exposure by zones.

Domains	Zones		
	Fear, *n* (%)	Learning, *n* (%)	Growth, *n* (%)
Relating to family and friends	137 (34.0%)	108 (26.8%)	158 (39.2%)
*COVID Encounters*			
No	40 (28.0%)	32 (22.4%)	71 (49.7%)
Positive outpatient	56 (41.2%)	38 (27.9%)	42 (30.9%)
Suspect, later negative	38 (32.2%)	38 (32.2%)	42 (35.6%)
Positive inpatient	3 (50.0%)	0 (0.0%)	3 (50.0%)
Relating to myself	47 (11.7%)	201 (50.0%)	155 (38.5%)
*COVID Encounters*			
No	15 (10.5%)	73 (51.0%)	55 (38.5%)
Positive outpatient	9 (6.6%)	74 (54.4%)	53 (39.0%)
Suspect, later negative	22 (18.6%)	50 (42.4%)	46 (39.0%)
Positive inpatient	1 (16.7%)	4 (66.7%)	1 (16.7%)
Relating to my practice/workplace	18 (4.5%)	330 (81.9%)	55 (13.6%)
*COVID Encounters*			
No	8 (5.6%)	114 (79.7%)	21 (14.7%)
Positive outpatient	3 (2.2%)	112 (82.4%)	21 (15.4%)
Suspect, later negative	5 (4.2%)	100 (84.7%)	13 (11.0%)
Positive inpatient	2 (33.3%)	4 (66.7%)	0 (0.0%)
Relating to the community	20 (5.0%)	329 (81.6%)	56 (13.9%)
*COVID Encounters*			
No	3 (2.1%)	121 (84.6%)	19 (13.3%)
Positive outpatient	8 (5.9%)	106 (77.9%)	22 (16.2%)
Suspect, later negative	7 (5.9%)	96 (81.4%)	15 (12.7%)
Positive inpatient	2 (33.3%)	4 (66.7%)	0 (0.0%)

**Table 3:** Results of questionnaire by area of practice.

Domains	Zones		
	Fear, *n* (%)	Learning, *n* (%)	Growth, *n* (%)
Relating to Family and friends			
Area of Practice			
Hospital	10 (28.6%)	10 (28.6%)	15 (42.8%)
Others	7 (26.9%)	4 (15.4%)	15 (57.7%)
Private community	107 (34.4%)	84 (27.0%)	120 (38.6%)
Public community	13 (41.9%)	10 (32.3%)	8 (25.8%)
Relating to Myself			
Area of Practice			
Hospital	3 (8.6%)	23 (65.7%)	9 (25.7%)
Others	2 (7.7%)	10 (38.5%)	14 (53.8%)
Private community	38 (12.2%)	155 (49.8%)	118 (38.0%)
Public community	4 (12.9%)	13 (41.9%)	14 (45.2%)
Relating to my Practice/workplace			
Area of Practice			
Hospital	1(2.9%)	28 (80.0%)	8 (17.1%)
Others	0 (0.0%)	20 (76.9%)	6 (23.1%)
Private community	14 (4.5%)	258 (83.0%)	39 (12.5%)
Public community	3 (9.7%)	24 (77.4%)	4 (12.9%)
Relating to the Community			
Area of Practice			
Hospital	1(2.9%)	24 (68.6%)	10 (28.6%)
Others	0 (0.0%)	24 (92.3%)	2 (7.7%)
Private community	15 (4.8%)	257 (82.6%)	39 (12.5%)
Public community	4 (12.9%)	22 (71.0%)	5 (16.1%)

**Table 4:** Overall zone distribution by age group and area of practice.

Domains	Zones			
	Fear, *n* (%)	Learning, *n* (%)	Growth, *n* (%)	Mixed, *n* (%)
Age group				
*N* = 403	5 (1.2%)	202 (50.1%)	29 (7.2%)	167 (41.4%)
≤34	1 (3.2%)	14 (45.2%)	1 (3.2%)	15 (48.4%)
35–44	1 (2.1%)	18 (37.5%)	2 (4.2%)	27 (56.3%)
45–64	3 (1.2%)	136 (57.2%)	23 (8.9%)	96 (37.2%)
≥65	0 (0.0%)	34 (51.5%)	3 (4.5%)	29 (43.9%)
Area of Practice				
Hospital	1 (2.9%)	20 (57.2%)	4 (11.4%)	10 (28.6%)
Others	0 (0.0%)	11 (42.3%)	5 (19.2%)	10 (38.5%)
Private Community	3 (1.0%)	159 (51.1%)	16 (5.1%)	133 (42.8%)
Public Community	1 (3.2%)	12 (38.7%)	4 (12.9%)	14 (45.2%)

## Data Availability

The data presented in this study are available on request from the corresponding author. The data are not publicly available due to institutional restrictions.

## Supplementary Materials

The following are available online at https://www.mdpi.com/article/10.3390/ijerph18189801/s1, Material 1: Survey Questions.
