# Supplementary material for: Mental Resilience of Medical Practitioners in Singapore during COVID-19: Survey Results from a Webinar Course on Resilience
Source: Int J Environ Res Public Health. 2021 Sep 17;18(18):9801. doi: 10.3390/ijerph18189801 (PMC8471345; doi:10.3390/ijerph18189801)
Supplement: Supplementary file 1 [file ijerph-18-09801-s001.zip › ijerph-1327877-supplementary.pdf]

Supplementary: Survey Questions

What zone am I in now?

Demographics:

1. How old are you?
    a. 34 and below
    b. 34 – 44 years
    c. 45 – 64 years
    d. 65 and above
2. Where do you practice?
    a. Private community
    b. Public community
    c. Hospital
    d. Others
3. Have you encountered any patient with COVID-19?
    a. No
    b. Yes, COVID suspect, later negative
    c. Yes, COVID positive, outpatient
    d. Yes, I look after inpatient COVID patients

'What ZONE AM I IN NOW during COVID-19'?

1. Relating to family & friends
    a. I feel safer to isolate myself from my family though I have used the appropriate PPE at work. **(fear zone)**
    b. I aim to use circuit breaker stay-home to improve bonding with my family. **(growth zone)**
    c. I keep the same contact with my friends within the restrictions imposed by circuit breaker. **(learning zone)**

2. Relating to myself
    (1) I accept what I cannot control. **(learning zone)**
    (2) I get upset when I think of the people who have aggravated the COVID situation. **(fear zone)**
    (3) I have discovered novel routines to enrich my life, despite the COVID outbreak. **(growth zone)**

3. Relating to my practice/ workplace
    a. I wish I could stop my medical practice till COVID-19 is over. **(fear zone)**
    b. I learn how best to protect myself and my staff. **(learning zone)**
    c. I counsel my colleagues/healthcare workers who are stressed and fearful. **(growth zone)**

4. Relating to the community
    a. I recognise that everyone is trying their best in these difficult times. **(learning zone)**
    b. I make my talents available to those who need them. **(growth zone)**
    c. I complain frequently relating to the policies implemented in this COVID period. **(fear zone)**
